# Modular Chip-Based nanoSFC–MS for Ultrafast Separations

**DOI:** 10.1021/acs.analchem.4c01958

**Published:** 2024-08-17

**Authors:** Chris Weise, Martin Schirmer, Matthias Polack, Alexander Korell, Hannes Westphal, Julius Schwieger, Rico Warias, Stefan Zimmermann, Detlev Belder

**Affiliations:** † University Leipzig, Linnestrasse 3, Leipzig 04103, Germany; ‡ UFZ Leipzig, Permoserstrasse 15, Leipzig 04318, Germany; § Leibniz University Hannover, Appelstrasse 9a, Hannover 30167, Germany

## Abstract

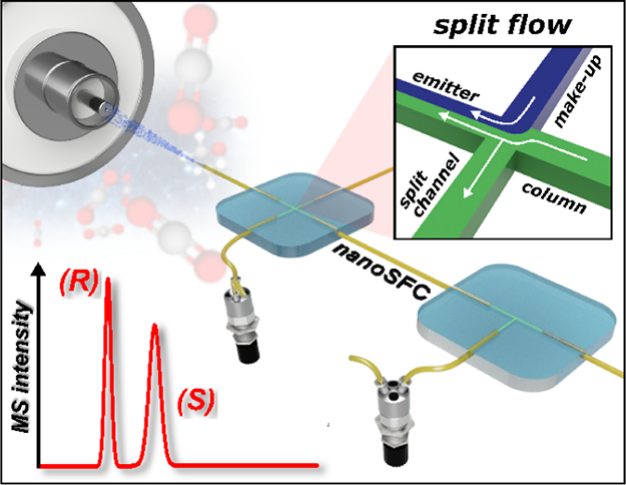

This study presents the development of a miniaturized device for supercritical fluid chromatography coupled with mass spectrometry. The chip-based, modular nanoSFC approach utilizes a particle-packed nanobore column embedded between two monolithically structured glass chips. A microtee in the pre-column section ensures picoliter sample loads onto the column, while a microcross chip structure fluidically controls the column backpressure. The restrictive emitter and the minimal post-column volume of 16 nL prevent mobile phase decompression and analyte dilution, maintaining chromatographic integrity during transfer to the atmospheric pressure MS interface. This facilitates high-speed chiral separations in less than 80 s with high reproducibility.

The pursuit of rapid and efficient chromatography has become increasingly important, particularly in the pharmaceutical and life sciences industries, where high-throughput methodologies are essential.^1−3^ Recent advancements have culminated in achieving analysis times below the one-minute threshold.^4−7^

In this context, supercritical fluid chromatography (SFC) using CO_2_ as a mobile phase has gained momentum. Under supercritical conditions (pc = 74 bar, Tc = 31 °C), CO_2_ offers increased flow velocities and reduces column backpressure compared to incompressible LC solvents.^8−10^ Its gas-like diffusivity results in a faster mass transfer with both normal and reverse-phase stationary phases, as it can be mixed with a polar organic modifier to adjust polarity.^11^

Regarding the kinetic benefits of supercritical CO_2_ in chromatography, the seamless integration of all SFC functionalities onto a single glass microchip is attractive because it offers the potential to further reduce analysis times and enhance productivity while minimizing sample consumption and space requirements.^12−14^

As part of the lab-on-a-chip technology, chromatographic microchips have proven to ensure a pressure-stable bonding, reliable world-to-chip interface, and active temperature control.^15,16^ These features have laid the groundwork for the technical implementation of using supercritical eluents on the chip, as demonstrated in a proof-of-principle study where a chipSFC was coupled with different fluorescence detection techniques to perform a chiral separation in less than 10 s.^17^

Due to their compressibility, the density and solubility of supercritical eluents can be controlled by adjusting pressure and temperature. This capability must be considered when coupling chipSFC with a detection system, as it can introduce the risk of compromising chromatographic integrity through eluent phase separation and analyte precipitation.^18^ To prevent these unwanted effects, backpressure control is required. The type of backpressure regulation used depends on the detection technique employed.

For detection techniques performed under high-pressure conditions, such as fluorescence detection, the mobile phase decompresses after detection. In these cases, backpressure control can be achieved by connecting conventional backpressure equipment to the end of the microchip. Instrumental setups with detection at atmospheric pressure or below are fundamentally different because the mobile phase decompresses prior to detection. Consequently, the effluent must pass through a pressure-stable and ideally adjustable restrictive element before reaching the detector interface. In traditional SFC setups, commercial backpressure regulators (BPRs) are used for this purpose. However, their high swept volumes make them unsuited for micro- and nano approaches, such as in chipSFC.^19−21^ Thermally controlled microfluidic BPRs with low nanoliter swept volume are potential alternatives.^22^ Other post-column approaches add a pressure-regulating liquid to the effluent before it flows into a restrictive interface.^23,24^ This strategy does not contribute to the post-column volume and allows to impact the spray conditions.^25^ However, in such a configuration, backpressure regulation and spray conditions cannot be adjusted independently, resulting in analyte dilutions.^26,27^

Implementing a post-column split is an alternative approach to address the large volume of a conventional BPR while retaining the ability to control the column backpressure and spray conditions independently.^26^ In this configuration, the effluent is divided between a backpressure-stabilized split channel and a restrictive MS interface, where it decompresses to atmospheric conditions. Additionally, a make-up stream can be flexibly added to control polarity and density in the restrictor, as well as droplet size and proton availability during the spray process.^28^

In conventional supercritical fluid chromatography–mass spectrometry (SFC–MS), the split-flow interface involves two consecutive tee junctions, one for make-up dosing and another dedicated to flow splitting.^29,30^

Inspired by this, we present a novel miniaturized approach to SFC-MS that combines capillary and chip-based microfluidics in a modular configuration. To achieve this, we utilize selective laser-induced etching (SLE) as a fabrication technique to create custom-made monolithic structures from fused silica glass.^31^ Our proposed SFC system incorporates a chip-based post-column microcross structure that integrates make-up dosing and flow splitting into a single structural element, thereby minimizing extra-column volume.

## Experimental Section

### Chemicals and Materials

Pressurized CO_2_ (purity grade N45) from Air Liquide (France) served as a mobile phase. As a mobile phase modifier and make-up solvent, methanol was purchased from VWR (Germany). Formic acid was used as a make-up additive purchased from Sigma-Aldrich (Germany). Water purified by smart2pure (TKA, Germany) was used. 7-Amino-4-methyl-coumarin (abbreviated c120) as a fluorescent probe was acquired from Sigma-Aldrich (Germany). The sample mixture for the test separation consisted of dl-α-tocopherol, ergocalciferol, nicotinamide, and pyridoxine, all purchased from Sigma-Aldrich (Germany). *R*/*S*-Warfarin for chiral separation was purchased from Sigma-Aldrich (Germany).

### Selective Laser-Induced Etching (SLE) Manufacturing and Layout of the Chip Modules

**Figure 1:**
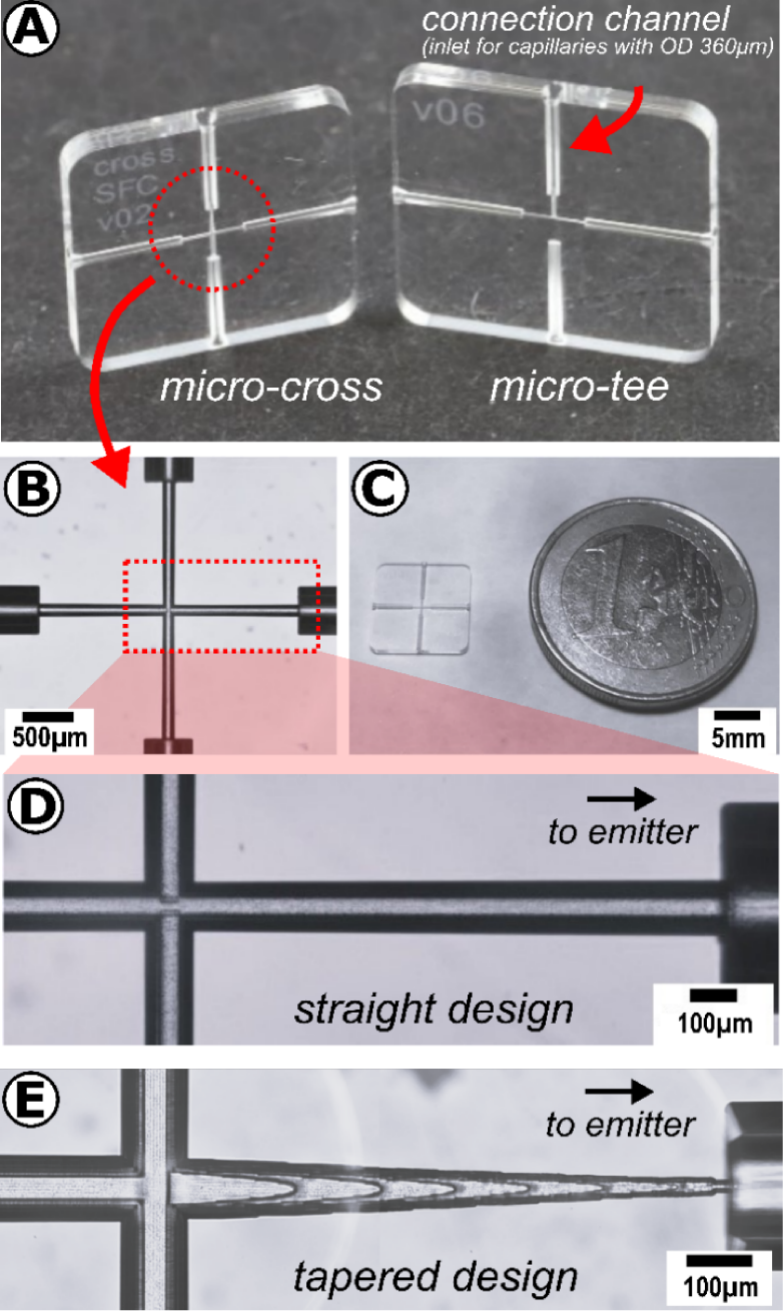
Photographic images of the SLE-manufactured glass-based chip modules used in this contribution. (A, C) Macroscopic view of chip modules with a microscopic insight view of (B) microcross structure. Insights of outgoing channel of microcross structure with (D) straight channel and (E) tapered channel design. All images were taken without the fused silica capillaries connected to the modules.

The developed modular SFC system consists of several components, including a nanobore column (OD 360 μm, ID 100 μm) connected to two microstructured chip modules made of fused silica glass. Figure 1A,C illustrates both chip modules and their spatial dimensions. The monolithic chip modules (length 10 mm, width 10 mm, depth 1 mm for microtee, 9 × 9 × 1 mm for microcross) were microstructured using selective laser-induced etching (SLE). The detailed procedure has been described in previous work.^32,33^ Briefly, the chip layout designed with computer-aided design (CAD) software was translated into machine code using computer-aided manufacturing (CAM) software (Alphacam R2 2017, Germany). To transfer the designs onto the 4-in. fused-silica wafer (1 mm thickness), SLE employed an IR laser (1030 nm Yb:YAG laser, pulse energy 230 nJ, pulse length 400 fs, feed rate 15.83 mm/s, layer distance *X*, *Y*-direction 2 μm, *Z*-direction 7 μm, 20× objective LHM-20×-1064, NA = 0). Subsequent etching of the laser-treated wafer in a hot KOH-based etching bath (8 mol/L, 85 °C; 22 h; avg. etching rate 230 μm/h) resulted in the desired high aspect-ratio channel structures. After etching, the remaining KOH was rinsed off with deionized water and the chip modules were dried with N_2_ for further processing. The SLE-manufactured chip modules featured a microfluidic structure at their center. Each channel in the microfluidic structure led to a 3–4 mm-long connection channel with an ID of slightly over 360 μm (Figure 1A). These connection channels enabled the chip module to connect to the necessary fluidic peripherals using glued-in fused silica capillaries (OD 360 μm, ID 100 μm, Molex, USA) with multicomponent epoxy adhesive (EPO-TEK 301, Epoxy Technology, USA). The ends of the fused silica capillaries were polished using a capillary polishing station (MS Wil, Netherlands) before gluing. One of the four connection channels was equipped with a fused silica capillary with reduced inner diameter (OD 360 μm, ID 10 or 20 μm, various lengths ranging from 4.5 to 80 cm), serving either as a capillary restrictor for fluorescence measurements or as a restrictive MS emitter during evaluation of the microcross chip module. Chip modules with three different microfluidic structures were used during this study. The chip module with a microtee structure (Figure 1A, right) served as a pre-column structure connected to the nanobore column head. The chip module with a microcross structure was attached to the back of the nanobore column and served as a post-column structure (Figure 1A, left and insights in Figure 1B). Both microstructures typically had straight channel geometry and a cylindrical channel cross-section with an inner diameter of 100 μm (Figure 1D). An improved microcross structure featured a tapered channel to reduce the post-column volume and provide a smoother pressure drop between the chip and the restrictive emitter. In this design, the inner diameter of the emitter channel tapered from 100 to 22.5 μm over a length of 1 mm (Figure 1E). The CAD design of the corresponding microcross is illustrated in Figure S1. For the final chip-based nanoSFC–MS experiments, a restrictive emitter (OD 360 μm, ID 10 μm, l 7 cm) was used. The blunt end of an emitter was tapered with lapping paper (grain size 2000, HPX, Belgium) to achieve a smaller surface area at the emitter tip.

### Manufacturing of Nanobore Column

A polyimide-coated fused silica capillary (OD 360 μm, ID 100 μm) served as a precursor for a nanobore column. The fused silica capillary of the desired length was connected to an HPLC pump-operated packing station (pressure limit at 450 bar) to introduce a slurry of stationary phase particles. An inlet filter with 1 μm pore size (VICI, Switzerland) was placed at the back of the column to retain the particle bed during packing. For achiral separation, the capillary column was packed with 5 μm silica-based 2-ethylpyridine particles (purchased as bulk material from Bischoff, Germany) using acetonitrile as an incompressible packing liquid. IG-3 particles with a size of 3 μm (purchased as bulk material from Daicel, Japan) were used for chiral separations. After packing with the desired stationary phase, the capillary was depressurized and dried at 60 °C overnight on a hot plate (VMR, Germany) to remove the residual solvent and ensure proper integration of the frit structures. Frit generation involved a sintering process in which both ends of the packed capillary were pulled through a butane flame (Proxxon, Germany).^34^ Afterward, the stability and flow resistance of the manufactured nanobore capillary column was tested by flushing with acetonitrile (*F* = 3 μL/min). Once tested, the column was ready for integration into the chip modules. Additional details, including a description of the slurry packing station, can be found in Figure S2.

### Fluidic Setup and Sample Injection

**Figure 2:**
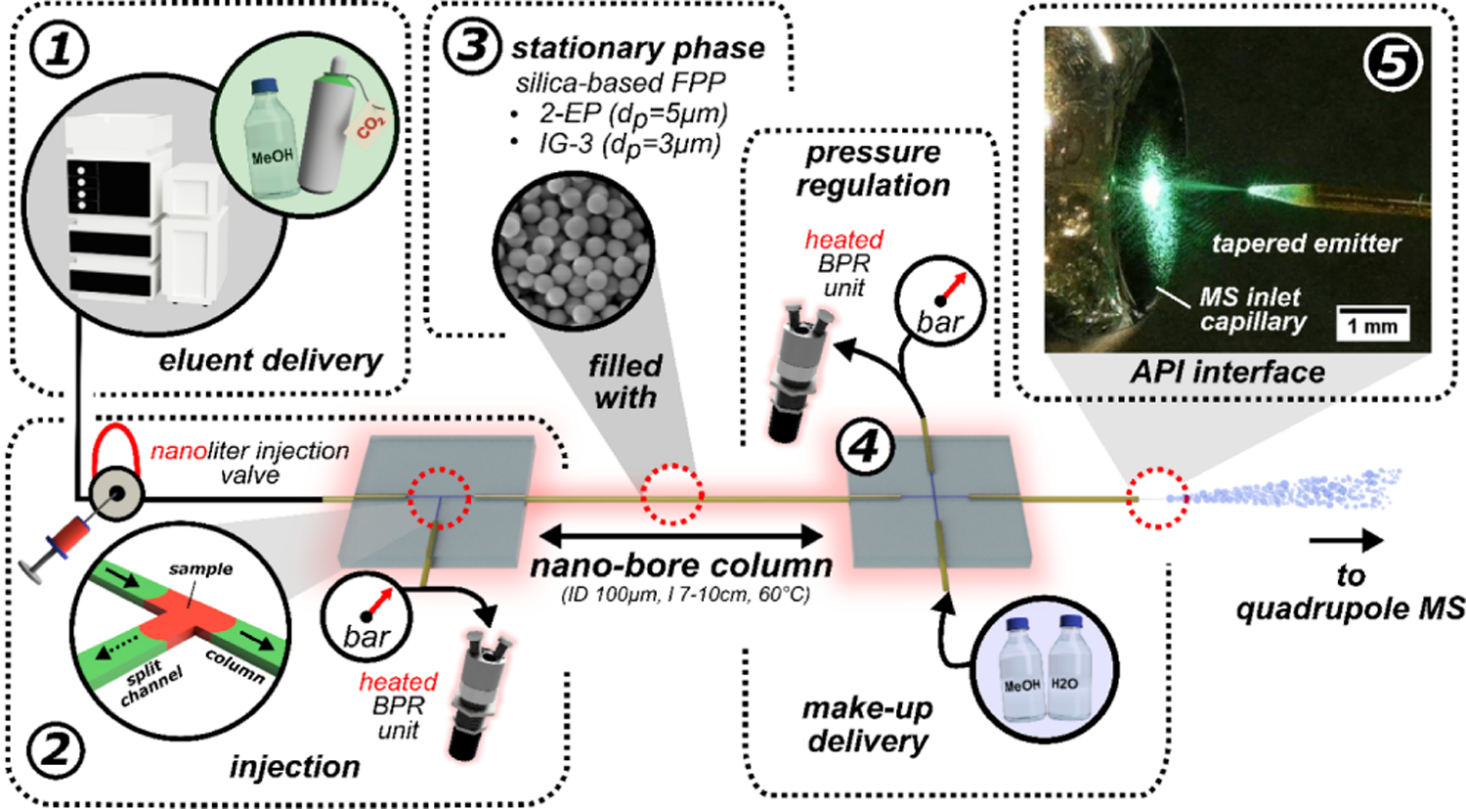
Schematic representation of the modular chip-based nanoSFC–MS setup. (1) SFC pump for eluent delivery, (2) chip module with microtee structure for sample injection, (3) packed nanobore column for separation, (4) chip module with microcross structure in the post-column section for pressure regulation and make-up delivery, and (5) restrictive emitter with a tapered tip for atmospheric pressure coupling to single quadrupole MS. Pressure sensors in the pre- and post-column sections monitor the pressure drop across the nanobore column. Detailed capillary dimensions are provided in Figures S3 and S4.

The developed modular chip-based nanoSFC system was connected to an external fluidic circuitry similar to those used in previous SFC investigations utilizing conventional, pressure-tight nano fittings (VICI Valco, Switzerland).^24^Figure 2 illustrates the experimental setup. Briefly, the circuitry was driven by a pressure-dependent SFC pump (1260 Infinity SFC System, Agilent Technologies, USA) that supplied the CO_2_-based eluent. An external nano injection valve (C74MPKH-4674, VICI, Switzerland) with a 5 nL sample loop was installed between the solvent delivery system and the modular nanoSFC system. By actuating the nano injection valve, a sample plug was inserted into the subcritical CO_2_-based eluent and transported to the microtee chip module. Here, the eluent underwent flow splitting between the nanobore column and the split channel, facilitating the loading of a picoliter fraction of the injected sample plug onto the column.^24^ To prevent phase separation during the flow splitting and adjust the pressure, a heated backpressure stabilization was connected to the chip modules in the pre- and post-column. It consisted of a heater for 1/16 stainless steel tubing (Caloratherm, Selerity Technologies, USA) and a dynamic SFC backpressure regulation unit (VICI, Switzerland). Both BPR units were heated to avoid damage from dry ice formation during decompression and submerged in IPA to prevent corrosive damage. An isocratic HPLC pump (LC-40, Shimadzu, Japan) ensured the delivery of the viscous make-up flow in the post-column chip module. An analog-to-digital converter, together with the Clarity Chromatography Software (Data Apex, Czech Republic), was used to trigger the switching of the nano injection valve, the start of the MS detection, and analog read-outs of pre- and post-column pressure sensors. The setup was automated for reproducibility evaluations to perform ten consecutive chipSFC–MS experiments within 15 min.

### Fluorescence Measurements

The proposed post-column chip module with the microcross structure was evaluated using a fluorescent sample (7-amino-4-methyl coumarin, c120, c = 500 μM dissolved in MeOH for the imaging, *c* = 100 μM for FLD measurements). Therefore, an inverted epifluorescence microscope (IX-71, Olympus, Japan) equipped with a short-arc mercury vapor lamp (Osram HBO 101W), a 40× lens (LUCPlanFLN, Olympus, Japan), an excitation filter (bandpass 350/50 nm), a dichroic mirror (at 380 nm) and an emission filter (long pass 390 nm) was used. The fluorescence signal was detected by a side-on photomultiplier tube (H9305-03, Hamamatsu). Data were recorded by Clarity Chromatography Software (Data Apex, Czech Republic). Details about the fluorescence instrumentation can be found in Figure S5.

### MS Measurements

For MS detection, the developed modular SFC assembly was placed onto a hot plate set at 60 °C (VWR, Germany), installed in front of the aperture of a single quadrupole mass spectrometer (6150B, Agilent Technologies, USA). The quasi-molecular ions of the achiral and chiral separations were detected in positive ion mode, using a capillary potential of −4.0KV at an acquisition speed of maximal 8 Hz. A detailed overview of the MS parameter and the isotopic masses of protonated analyte ions can be found in Figure S10. During the measurements, the post-column chip model was observed from the top using a monochrome camera (AxioCam 503, Zeiss, Germany) equipped with a lens (10×, Z6 APO, Leica). A digital microscope (10×, Andonstar, China) was used to visually inspect the MS interface’s lateral view.

## Results and Discussion

Coupling chip-based supercritical fluid chromatography (chipSFC) with ambient pressure ionization is technically challenging because the supercritical effluent must be depressurized to atmospheric conditions.^18^ During this process, supercritical CO_2_ expands into a gas, reduces its solubility, and can disrupt chromatographic integrity.^23^ To manage the decompressing effluent, a post-column split-flow offers a viable solution because it controls column backpressure while minimizing band dispersion. These benefits are achieved by splitting the effluent stream and directing one fraction to a backpressure regulator. The remaining effluent fraction flows directly to the detector and is decompressed by a restrictor along the way. To counteract analyte precipitation during this process, an incompressible make-up liquid is added.^25^ Since flow splitting and make-up flow addition can be elegantly combined within a cross-structure, it is ideally suited as a post-column split-flow interface for chip-based SFC–MS coupling.

To meet the requirements for a chip-based MS interface for supercritical effluents we used the SLE technology to manufacture a low-volume, glass-based chip module with a custom-designed microfluidic cross (Figure 1). The chip module contains four lateral cavities for glued-in capillaries (OD 360 μm, ID 100 μm) to ensure a pressure-stable connection to the two inlets and outlets. The nanobore column and make-up pumping systems are connected to the inlets, while the other two cavities serve as outlet channels for the split and emitter flows. Both outlets were backpressure stabilized to prevent CO_2_ decompression within the cross-structure. A BPR with an adjustable spring-loaded membrane was connected to the split flow outlet and a restrictive capillary (ID 20 μm, *l* = 80 cm) was connected to the emitter outlet. The described microcross chip module was integrated into a fluorescence setup for preliminary experiments. The detailed instrumental setup is shown in Figure S5.

### Evaluation of the Microcross Chip Module

**Figure 3:**
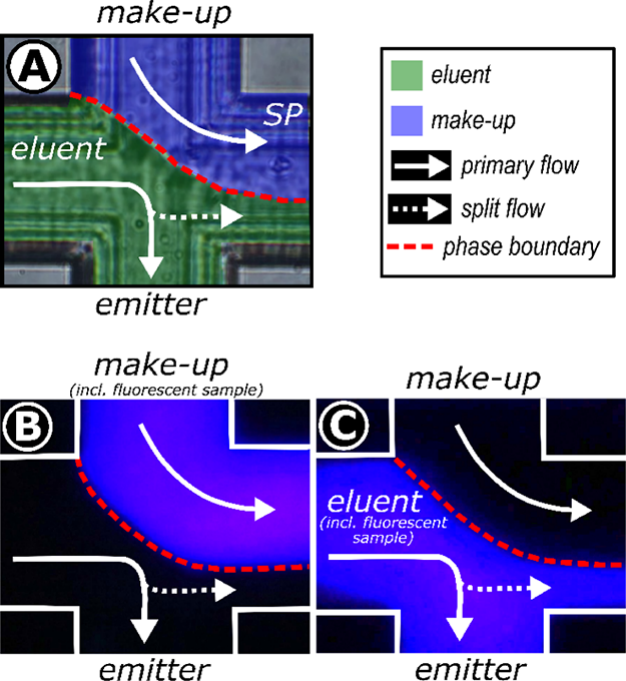
Visual and fluorescence examination of the microcross chip module (A) Microscopic images of the microcross chip module showing the phase boundary between the incoming primary (eluent) stream (*F* = 1 mL/min, inlet pressure = 120 bar, 50:50 v/v CO_2_:MeOH, room temperature) and the make-up stream (*F* = 8 μL/min, 90:10 v/v MeOH:H_2_O, 0.1% FA). Injections of a fluorescent sample plug (7-amino-4-methyl-coumarin (c120), *c* = 500 μM dissolved in MeOH) (B) into the make-up stream and (C) into the primary (eluent) stream to visualize the mass transfer within the microcross chip module. Cross configuration: eluent 90° to make-up, eluent 180° to split channel, SP stands for split channel.

In contrast to metal or polymeric connection technologies, the glass-based chip modules offer excellent optical transparency, enabling microscopic and fluorescence-based inspections to evaluate the flow behavior, sample transfer integrity, and splitting performance within the microcross structure. For this purpose, the chip module was exposed to a CO_2_ stream and a methanolic make-up stream that converged perpendicularly before being split into the emitter and split channels. Due to the density differences between the two streams, a phase boundary is visible, indicating a laminar flow regime (Figure 3A).

A fluorescent sample plug was injected separately into each stream using a nano injection valve to investigate a possible sample transfer across the phase boundary. The microscopic data revealed that the fluorescent probe remained within its injected stream, as exemplified in the respective images in Figure 3B,C.

**Figure 4:**
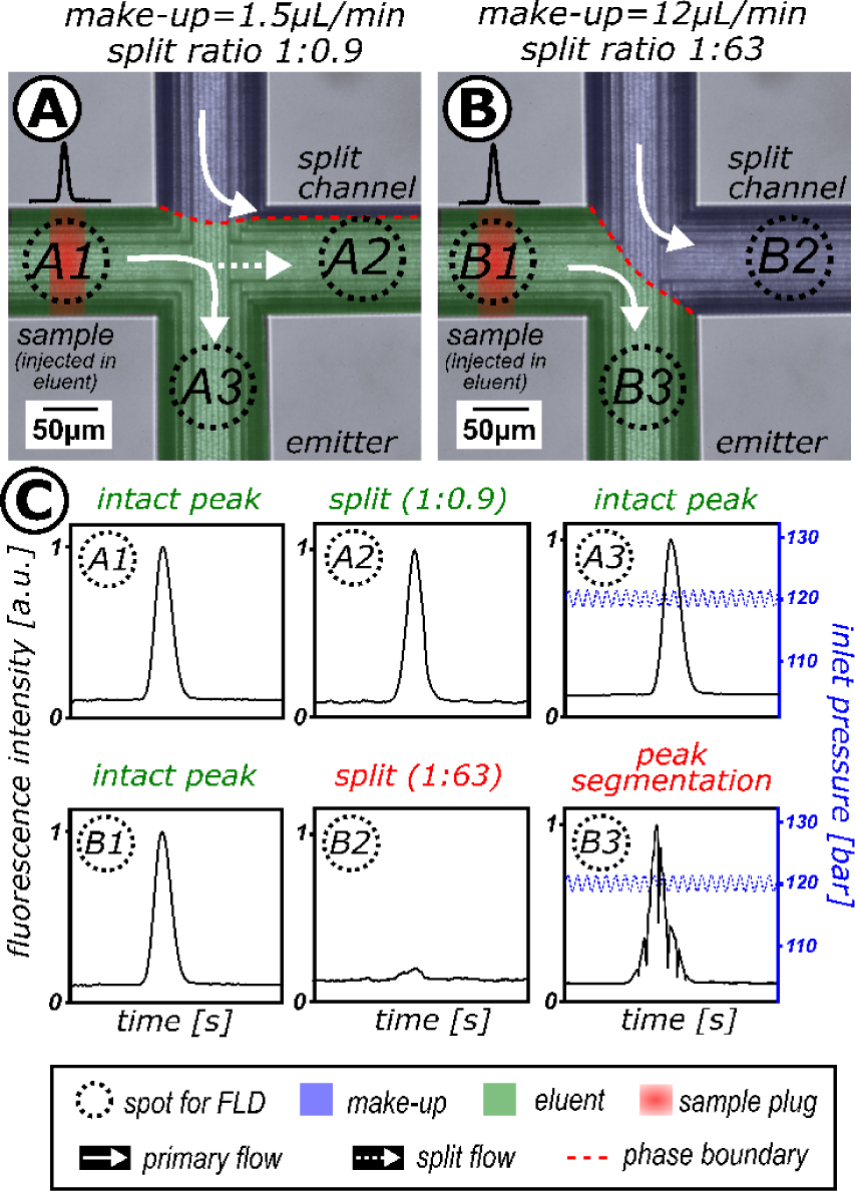
Inspecting the split behavior of the microcross chip module. Microscopic view of the microcross under different make-up flow conditions: (A) 1.5 μL/min, split channel open, split ratio 1:0.9 and (B) 12 μL/min, split channel temporarily closed, split ratio 1:63. Numeric labels indicate the positions for the fluorescence detection during signal tracing (7-amino-4-methyl-coumarine (c120), *c* = 100 μM dissolved in MeOH). (C) FLD chromatograms acquired at the inlet (A1/B1), emitter (A2/B2), and split channel (A3/B3) illustrate the differences in signal integrity at the emitter channel. Instrumental parameter: primary flow: 50:50 v/v CO_2_:MeOH eluent, 120 bar, make-up: 90:10 v/v MeOH:H_2_O, restrictor: ID 20 μm, length 80 cm, no column.

Due to differences in compressibility between the primary and make-up streams, the signal integrity of the sample plug can be compromised when passing through the microcross chip module. To investigate this, the analyte signal was tracked by fluorescence at three distinct points within the chip module (Figure 4A). The corresponding chromatograms exhibit intact signal peaks before and after the split, confirming the uncompromised transfer of the sample plug through the chip module (Figure 4C position A1 to A3).

As the primary flow is split, only a portion of the sample plug reaches the MS interface and is detected. Therefore, understanding the splitting behavior of the microcross structures is crucial. For this purpose, a fluorescent sample was injected under varying splitting conditions by increasing the make-up flow from 1.5 μL/min (Figure 4A) to 12 μL/min (Figure 4B). The corresponding split ratios were determined by comparing the detected peak areas in the split channel (position A2 or B2) to the peak areas detected before the split (position A1 or B1). The results, shown in Figure S6, indicate that the split ratio decreases from 1:0.9 to 1:63 as the make-up flow increases. The decreasing split ratio is attributed to the incompressible make-up flow constricting the split channel (Figure 4B). Consequently, the compressible sample-containing primary stream is displaced into the emitter channel, mimicking a splitless interface. The (temporary) closure of the split channel causes the segmentation of signal peaks synchronized with the pressure fluctuations of the eluent feed pump (Figure 4C position B3). This behavior can be explained by the absence of pressure damping since the make-up flow prevents access to the back-pressure regulator. Experiments using a true splitless interface with a make-up stream via a microtee chip module (Figure S7) confirmed this. An appropriate flow restrictor between the SFC pump and the chip module, such as a column structure or a dynamic check valve, can mitigate this unwanted behavior in the current setup.^21^

### Investigations Regarding MS Sensitivity

The results of the fluorescence measurements using the developed SLE-fabricated microcross chip module raise the question of whether the intact peak signal can withstand the decompression within the restrictor to be detected by atmospheric pressure ionization mass spectrometry. Unsuitable restrictors that lead to premature decompression can result in the detection of segmented ion chromatograms, as gas bubbles segment the analyte band.^35^

To investigate this, we installed an 80 cm long fused silica capillary, which served as a restrictor in front of the MS inlet capillary (set at 4KV). With this arrangement (Figure S8), a jet of effluent vapors directed toward the MS inlet could be generated out of 90:10 v/v CO_2_:MeOH mobile phase at 120 bar (Figure 2). Subsequent injections of dl-α-tocopherol (*V* = 5 nL, *c* = 100 μM) were detected as [M + H]^+^ and resulted in unsegmented ion chromatograms.

**Figure 5:**
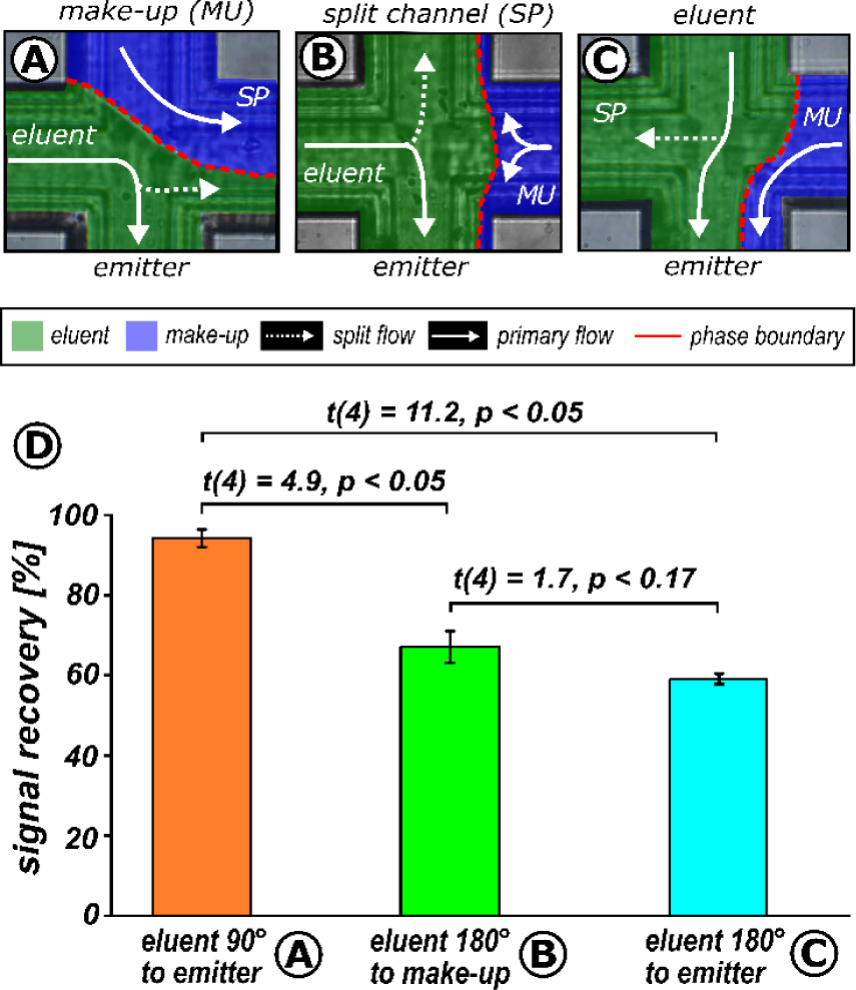
MS sensitivity of the microcross chip module. The MS performance of three different fluidic configurations: (A) eluent 90° to the emitter, (B) eluent 180° to the make-up, and (C) eluent 180° to the emitter are compared in (D). MS sensitivities were based on the signal recovery of three consecutive dl-α-tocopherol injections (*c* = 100 μM, including 1 mM ergocalciferol as an internal standard). The relative signal recovery [%] was calculated from the ratio of the peak area of both mixture compounds. The corresponding calibration curve is shown in the Figure S10). Results of an unpaired *t* test are shown for all combinations. Instrumental parameter: mobile phase 90:10 v/v CO_2_:MeOH, inlet *p* = 120 bar, no column was used. MS parameter: positive ion mode, SIM scans recorded at 473*m*/*z* (for dl-α-tocopherol as [M + H]^+^) and 397.5*m*/*z* (for ergocalciferol as [M + H]^+^).

The ionization mechanisms and complex processes occurring during decompression remain subjects of significant debate within the scientific community.^36−40^ To provide further insights, we have investigated how mass flow and the composition of the flow exposed to the restrictive mass spectrometry (MS) emitter influence MS sensitivity. These investigations were conducted using a chip-based microcross module, operable in three distinct orientations (Figure 5A–C). Each orientation differs based on the presence of the make-up liquid in the emitter. To ensure consistency in our measurements, a fixed concentration of ergocalciferol (*c* = 1 mM) was added to a dl-α-tocopherol sample mixture as an internal standard. This compensated for instrumental variables, like emitter position, by normalizing the detected peak areas of the analyte ion chromatogram. As the resulting ratios between the analyte and standard could be correlated to a specific dl-α-tocopherol concentration (Figure S9), it was possible to determine how much of a 100 μM dl-α-tocopherol sample could be detected by the MS. This was expressed as signal recovery in %. A signal recovery of 100% means that the entire amount injected was detected during the MS scan.

Figure 5D compares the MS response of the three fluidic orientations (eluent 90° to emitter referred to as A, eluent 180° to the make-up channel as B, and eluent 180° to the emitter as C). Herein, orientations directing no make-up flow into the MS interface show higher signal recovery (93.7 ± 1.9% in Figure 5A) than orientations with a make-up portion present within the MS emitter (66.7 ± 3.8% in Figure 5B and 58.8 ± 1.3% in Figure 5C).

The results are consistent with the fluorescence data. This suggests, that as the stream entering the split channel becomes less compressible (due to the addition of the make-up flow), more sample mass flow is directed into the MS interface. Transferring a more incompressible effluent in the MS is likely to result in a poorer ionization efficiency, as nebulization and droplet size are negatively affected by the higher surface tension.^27^

Although the addition of the make-up stream reduced the MS sensitivity under the conditions presented, it is still critical. Especially when the eluent contains lower amounts of modifier, the make-up stream adjusts the density of the effluent and prevents the precipitation of analytes in the MS restrictor. This has proven effective in long-term operation as it avoids clogging and replacing the glued-in restrictive MS emitter. Therefore, the orientation of the interface shown in Figure 5C was chosen for the subsequent SFC–MS experiments.

**Figure 6:**
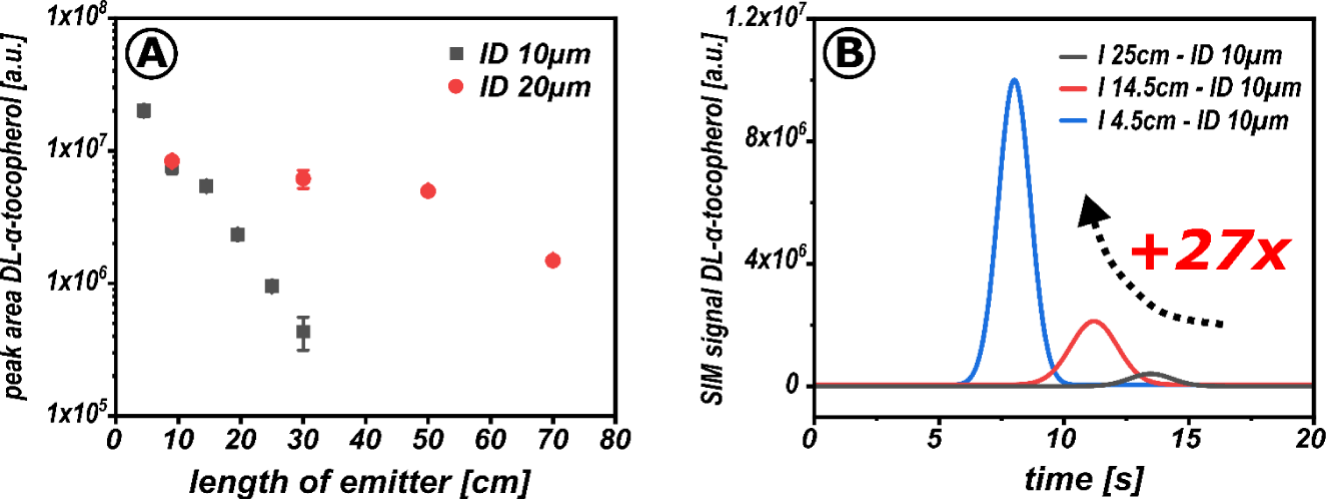
Assessment of the MS sensitivity of the microcross chip module under different mass flow conditions. The geometry of the given capillary restrictor was adjusted to set different mass flows. (A) Impact of restrictor length and inner diameter on MS response of dl-α-tocopherol [M + H]^+^ 473 *m*/*z*. (B) Overlay of SIM chromatograms of dl-α-tocopherol [M + H]^+^ 473 *m*/*z* for different lengths of an ID 10 μm restrictor. Mobile phase 90:10 v/v CO_2_:MeOH, inlet *p* = 120 bar, no column was used, make-up: 1.5 μL/min 90:10 v/v MeOH:H_2_O, 0.1% FA, microcross configuration eluent 180° to the emitter (Figure 5C).

In a series of experiments, the length of the restrictive emitter was shortened to adjust the mass flow to the MS. The effect on the peak area is shown in Figure 6. A 27-fold increase in peak area with decreasing emitter length illustrates the advantage of this adjustment (Figure 6B) and confirms the mass-flow-dependent MS sensitivity of the developed interface.

The increased sensitivity is not entirely due to the higher mass flow rate, as indicated by the differences in sensitivity between the ID 10 μm and ID 20 μm emitters (Figure 6A). The pressure drop and its steepness also play a role, influencing flow velocity and decompression time course. These findings suggest that overcoming a given pressure drop as quickly as possible is advantageous.^41^ Although the split-flow interface has a lower MS sensitivity, it offers the great practical advantage that the column back pressure can be precisely adjusted. This makes method development more straightforward and improves reproducibility. In addition, robustness is increased because sample precipitation in the restrictor is prevented.

### Development of a Modular Chip-Based nanoSFC–MS System

After the series of preliminary experiments demonstrating the MS compatibility of the developed microcross chip module, the next logical setup was its integration into a miniaturized SFC system. Developing a chromatographic system requires the pressure-tight implementation of an injection and column compartment to the evaluated post-column microcross structure. Previous chipSFC studies have shown that the split injection principle delivers a picoliter sample plug to the column.^24^ Therefore, a second SLE-fabricated chip module was used for that purpose. The chip module features a microtee structure (Figure 1A, right chip module, channel ID 100 μm) to which the column and a heated BPR were connected. The backpressure-stabilized side channel of the microtee chip module generates the necessary pressure drop between the nanoinjection valve and the column, which enables the rapid transport of the sample plug. During this transfer, one fraction of the sample-containing eluent is split into the side channel, while the remaining fraction is loaded onto the heated column for subsequent SFC analysis.

Conventional fused-silica capillaries (OD 360 μm), flexible in length, with an ID of 100 μm, packed with a 2-ethylpyridine stationary phase served as nano column for achiral separations.^42,43^

The postcolumn microcross structure in this novel modular chip-based nanoSFC system was equipped with a 7 cm-long capillary emitter with an inner diameter of 10 μm. Using such a restrictor resulted in an abrupt constriction in the internal diameter (from 100 to 10 μm) between the microcross structure outlet and the emitter capillary entrance (Figure 1D, straight design). To smooth this transition, the design of the emitter channel in the chip module was changed from a cylindrical shape to a tapered shape (Figure 1E). Compared to the straight (linear) design, the tapered emitter channel design increases the overall pressure drop but reduces the inner diameter of the targeted outlet to 22.5 μm (Figure S1). Both adjustments, using smaller emitter dimensions and the tapered emitter channel, reduced the post-column volume of the developed interface to approximately 16 nL. In the final step, the three modules, the microtee chip device for sample injection, the packed nanobore column, and the optimized microcross chip module were joined together with epoxy resin (Figure S4). The assembled device was able to withstand inlet pressures of up to 220 bar. At such pressures, the glued interface between the chip module and connection capillaries tended to fail, although the chip modules themselves remained intact. This indicates that even higher pressure stability could be achieved with a more robust connection method, such as alternative adhesives or, preferably, laser-welded connections, as demonstrated by Lotter et al.^16^

The functionality of the whole device was evaluated by on-column injections of a dl-α-tocopherol. Using a 90:10 v/v CO_2_:MeOH mobile phase at 140 bar, the analyte eluted after 13 s (Figure S10). Subsequent measurement of a dilution series showed that the nanoSFC–MS could detect concentrations in the single-digit micromolar range, corresponding to an on-column load of less than 155 fg dl-α-tocopherol (only 7.2% of the injected 5 nL sample plug are loaded).^24^

### Application

**Figure 7:**
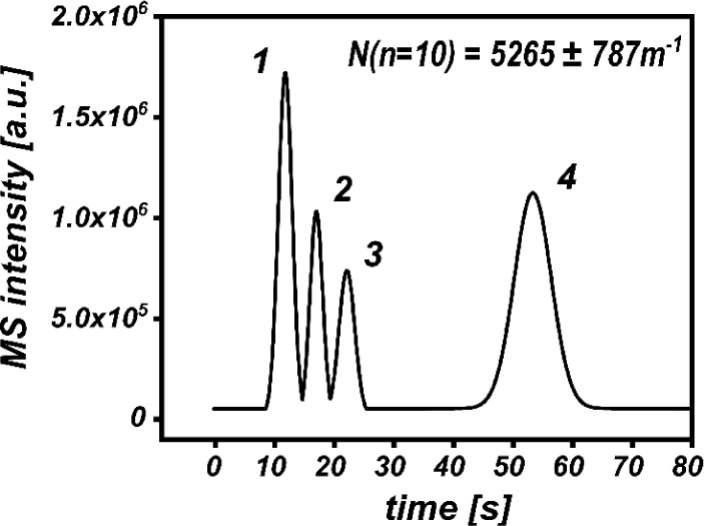
nanoSFC of fat- and water-soluble vitamins, sample: (1) dl-α-tocopherol (α-TOCO, 473 *m*/*z*, fragmentor: 300 V), (2) ergocalciferol (ERGO, 397.5 *m*/*z*, 275 V), (3) nicotinamide (NICO, 123 *m*/*z*, 200 V), and (4) pyridoxine (PYR, 170 *m*/*z*, 200 V) dissolved in MeOH, eluent: 90:10 v/v CO_2_:MeOH, column: 8 cm, 2-EP, dp = 5 μm, *t* = 60 °C, inlet *p* = 200 bar, outlet *p* = 110 bar, cross configuration: eluent 180° to the emitter, make-up: 1.5 μL/min 90:10 v/v MeOH:H_2_O, 0.1%FA, emitter: tapered, 7 cm, ID = 10 μm, MS parameter: BPC of all for [M + H]^+^ at 4 Hz, separation efficiency (*N*) was calculated based on pyridine, peak smoothing was applied, chromatograms generated during modifier optimization are found in Figure S11E.

After the initial injections confirmed the functionality of the modular chip-based nanoSFC, its analytical potential was explored. Supercritical CO_2_, with polarity similar to hexane and compatibility with polar solvents, offers a sustainable, cost-effective, and safe alternative to normal-phase chromatography, especially when coupled to ambient ionization mass spectrometry. Traditional normal-phase MS interfaces suffer from poor mixing and spray instabilities due to polar make-up addition. A CO_2_-based mobile phase reduces these issues and additionally offers self-nebulization.^44^ This was demonstrated by separating four water- and fat-soluble vitamins within 70 s using a 90:10 v/v CO_2_:MeOH mobile phase at 200 bar and 60 °C (Figure 7, chromatographic features in Table 1). When trying to further increase the resolution of the first three peaks, it was found that minor pressure drops across the column increase the resolution but also prolong the analysis time (Figure S11A–C). The calculated efficiencies indicate that the system is not running at the flow optimum (Figure S11D), but due to the split-flow design, it is difficult to determine the exact flow rates. One option to further increase the efficiency would be the use of sub-2 μm particles, but their application may require adapting the current packing procedure (Figure S2), which is limited to 450 bar.^45^

**Table 1:** Chromatographic Features of Vitamin Model Mixture

*n* = 10	retention time [s]^a^	fwhm [s]^a^	*R*_S_^a^
α-TOCO(1)	11.74 (0.8%)	2.64 (3.7%)	-
ERGO(2)	17.46 (0.8%)	2.75 (4.0%)	1.25 (4.7%)
NICO(3)	22.34 (1.3%)	2.62 (6.9%)	1.08 (8.7%)
PYR(4)	53.66 (1.3%)	6.20 (7.0%)	4.83 (7.0%)

a Results presented as mean (RSD in %) of ten measurements.

**Figure 8:**
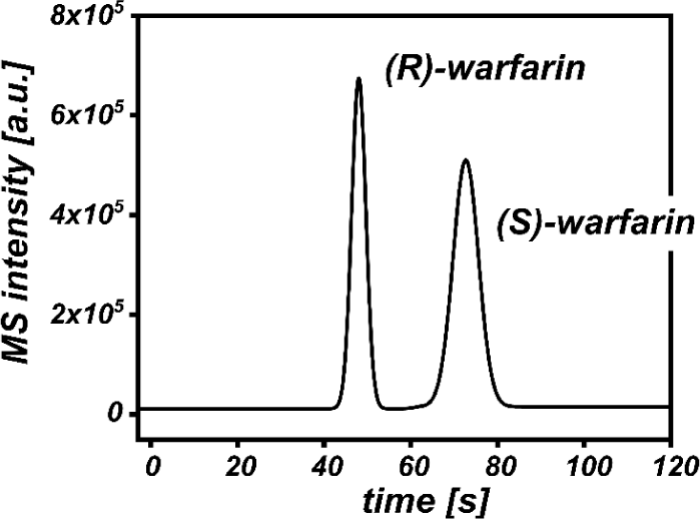
Chiral separation of racemic mixture of warfarin, sample: 500 μM warfarin dissolved in MeOH, eluent: 65:35 v/v CO_2_:MeOH, column: 10 cm, IG-3, dp = 3 μm, *t* = 60 °C, inlet *p* = 220 bar, outlet *p* = 85 bar, make-up: 1.5 μL/min 80:20 MeOH:H_2_O, 0.1%FA, emitter: tapered, length 7 cm, ID = 10 μm, MS parameter: SIM scan of 309 *m*/*z*, [M + H]^+^, fragmentor 200 V, 8 Hz, peak smoothing was applied.

In addition to the vitamin analysis, we investigated the use of a chiral column for the fast separation of enantiomers. For this purpose, a capillary column packed with the commercial chiral IG-3 material was assembled with two microfluidic chips, similar to the nonchiral column setup. This configuration allowed us to separate a mixture of *R*- and *S*-warfarin (detected as [M + H]^+^= 309 *m*/*z*) within 80 s (Figure 8). By automating sample injection and data acquisition, the chip-based nanoSFC–MS system could leverage fast equilibration and analysis times to perform multiple consecutive measurements. The acquired chromatogram series exhibited reproducible retention times (RSD < 1% in Table 2).

**Table 2:** Chromatographic Features of Warfarin Mixture

*n* = 6	retention time [s]^a^	fwhm [s]^a^	*R*_S_^a^
(*R*)-warfarin	46.5 (0.7%)	3.6 (3.6%)	-
(*S*)-warfarin	72.1 (0.4%)	6.6 (6.8%)	2.9 (6.2%)

a Results presented as mean (RSD in %) of six measurements.

## Conclusion

This study demonstrates the development of a miniaturized, supercritical fluidic chromatography system coupled with an ambient pressure ionization mass spectrometer. This chip-based nanoSFC is a modular analytical system that combines the advantages of chip- and capillary-based microfluidics. It comprises a nanobore column precisely integrated into two void-volume-free chip modules fabricated by selective laser etching. In the pre-column area, a microtee chip module ensures precise sample injection, while a microcross chip module after the column enables coupling with atmospheric pressure ionization mass spectrometry via a micro split flow strategy. This split-flow interface controls the column back pressure, maintains chromatographic integrity, and influences the decompression process of the mobile phase within the restrictive emitter and the spray conditions through a make-up flow. The applicability of the new design was demonstrated by rapid separations of vitamins and enantiomers within seconds. Future developments will focus on instrumental developments to further increase speed, separation efficiency, and sensitivity. Once these improvements are achieved, this technology will be applied to samples with complex matrices.

## Supporting Information Available

Detailed information about the dimensions of the SLE-fabricated microcross chip module, nanobore column fabrication, instrumentation and microfluidic setups used; fluorescence data including the evaluation of the microtee and split behavior of the microcross chip module; calibration curves, MS sensitivity data, and the effect of pressure drop and modifier on the achiral separation (PDF)
