# Supplementary material for: Modular Chip-Based nanoSFC–MS for Ultrafast Separations
Source: Anal Chem. 2024 Aug 17;96(34):13888–96. doi: 10.1021/acs.analchem.4c01958 (PMC11359387; doi:10.1021/acs.analchem.4c01958)
Supplement: Supplementary file 1 — ac4c01958_si_001.pdf [file ac4c01958_si_001.pdf]

SUPPORTING INFORMATION

Modular chip-based nanoSFC-MS for ultra-fast separations

Chris Weise[a], Martin Schirmer[b], Matthias Polack[a], Alexander Korell[a], Hannes Westphal[a], Julius Schwieger[a], Rico Warias[a], Stefan Zimmermann[c] and Detlev Belder*[a]

[a]Institute of Analytical Chemistry, University Leipzig, Linnéstrasse 3, 04103 Leipzig, Germany, [b] UFZ Leipzig, Permoserstrasse 15, 04318 Leipzig, [c] Leibniz University Hannover, Appelstrasse 9a, 30167 Hannover

Corresponding author:

*E-mail Detlev Belder: belder@uni-leipzig.de

Table of content

Figure S1 – Dimensions of SLE-fabricated micro-cross (tapered design) page S-2

Figure S2 – Packing procedure for nano-bore column page S-3

Figure S3 – Detailed overview of the fluidic setup page S-4

Figure S4 – Photographic representation of modular chip-based nanoSFC page S-5

Fluorescence experiments

Figure S5 – Fluorescence setup for evaluation of micro-cross chip module page S-6

Figure S6 – Split behavior of the micro-cross chip module page S-7

Figure S7 – Evaluation of micro-tee chip module page S-8

Mass Spectrometry experiments

Figure S8 – MS setup used for evaluation of the micro-cross chip module page S-9

Figure S9 – Suitability of the internal standard for determining MS sensitivity page S-10

Figure S10 – MS sensitivity of modular chip-based nanoSFC page S-11

Figure S11 – Influence of pressure drop and modifier on achiral separation page S-12

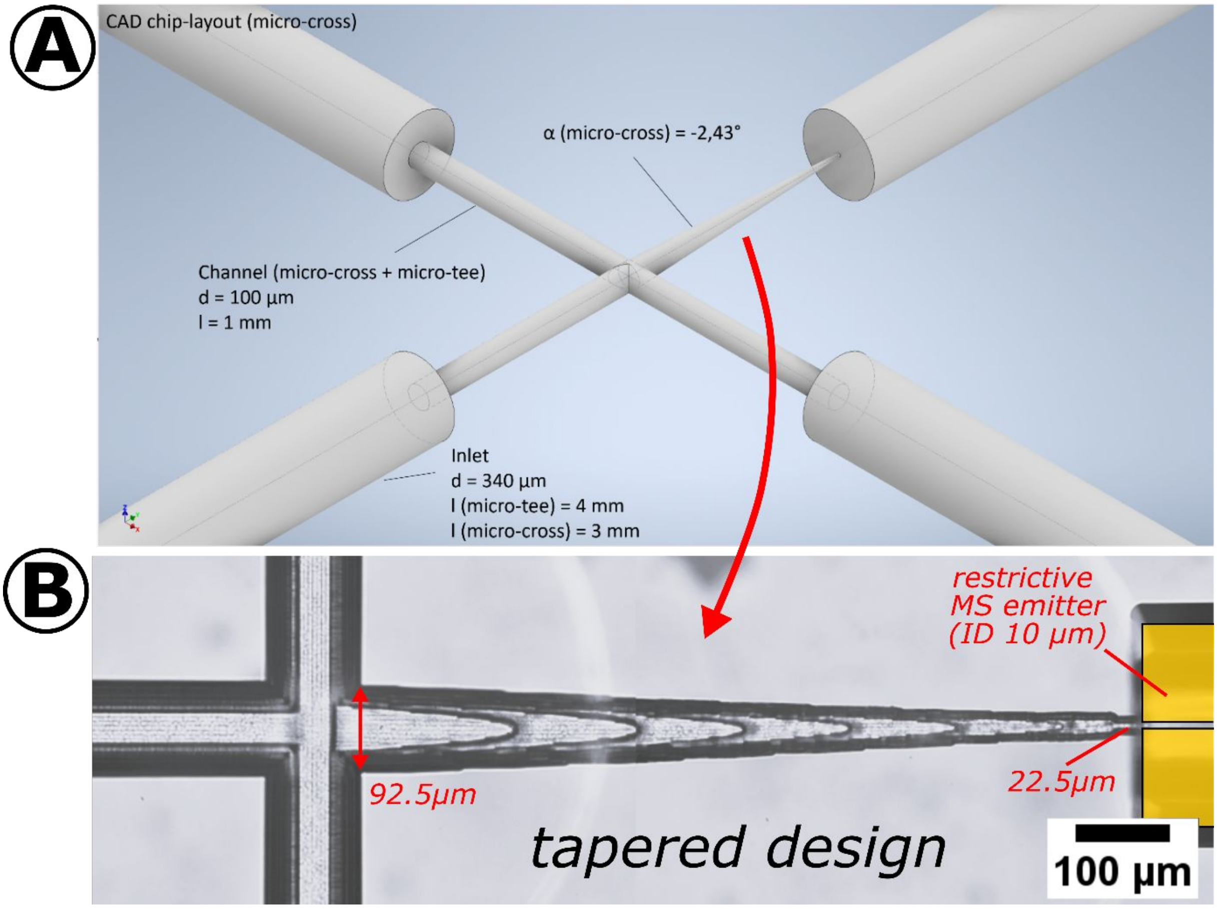

Figure S1 - Dimensions of SLE-fabricated micro-cross (tapered design). (**A**) CAD of the micro-cross (tapered design). The CAD considered the etch rate of pristine fused silica material (~ 1 µm/h) for dimensional accuracy. (**B**) The photographic image of the micro-cross after the SLE process allows determination of the accurate dimensions of the tapered channel.

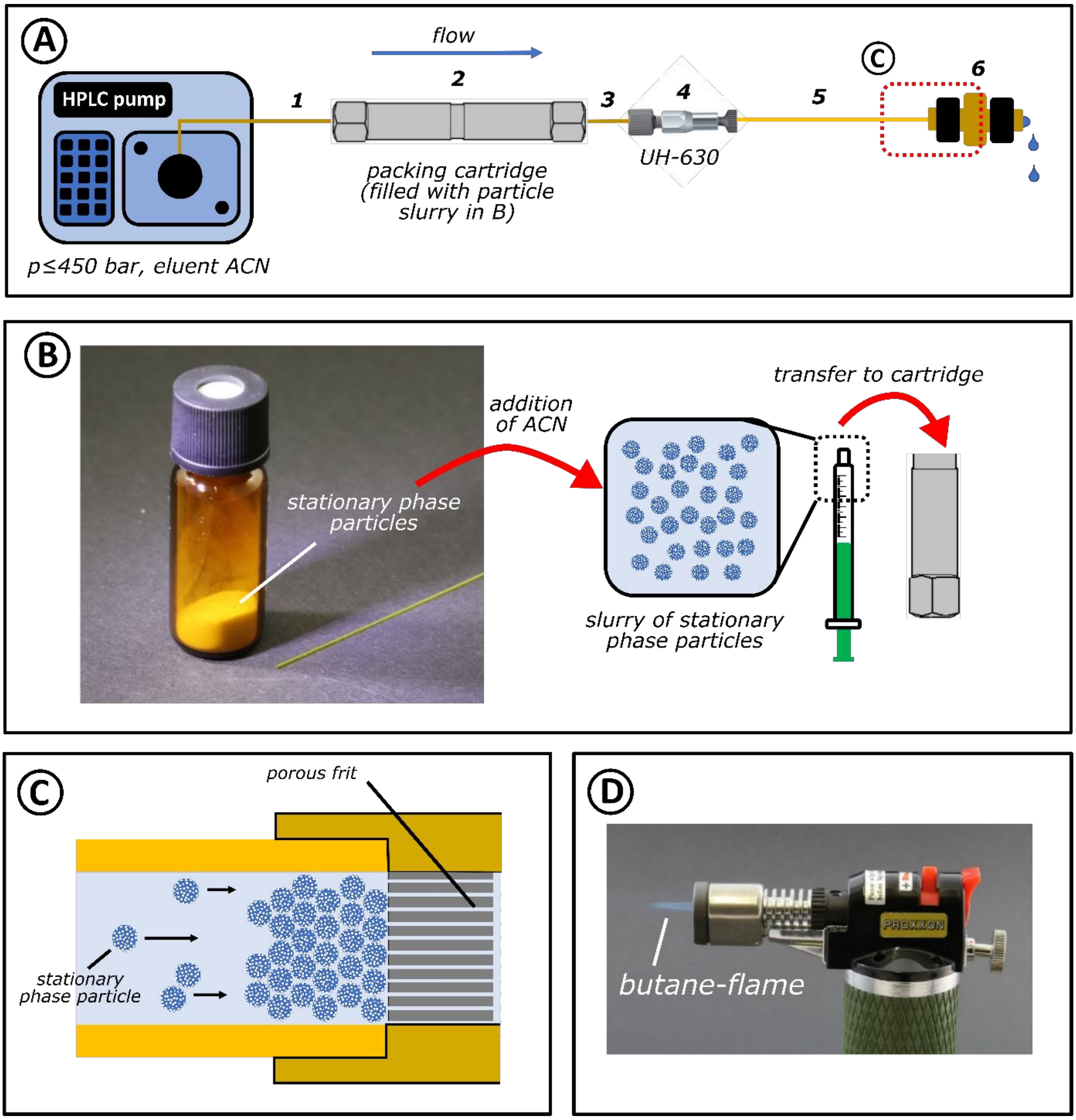

Figure S2 – Packing procedure for nanobore column – (**A**) Instrumental setup used for slurry packing of nanobore column. The setup is driven by a flow-dependent HPLC pump connected to a fluidic circuitry to pack a fused silica capillary with ID of 100µm (5). The dimensions of the consisting parts are listed in the following (1) PEEK capillary, OD 1/16, ID 200µm, (2) stainless steel column cartridge (2-piece, screwable, ID 1cm with OD 1/16 with 10-32 threads on both sides, (3) PEEK capillary, OD 1/16, ID 130µm, (4) UH-630 MicroTight adapter for OD 1/16 to 360µm, (5) Fused Silica capillary, OD 360µm, ID 100µm; (6) IDEX M-530 Mini MicroFilter Assembly equipped with M-133 SST filter (pore size 1 µm) (**B**) Prior to the packing procedure the column cartridge is filled with particle slurry (c=1-2mg/mL in ACN). (**C**) During the packing procedure, the particles (dp= 3-5µm) are retained by the porous steel frit (pore size 1µm). (**D**) After the fused silica capillary (5) is packed to the desired length, it is disconnected from the setup and dried on a hot plate (VMR, Germany) at 50°C overnight. Pulling both ends of the fused silica nano pre-column through a butane flame generates a porous frit based on the sintered particles.

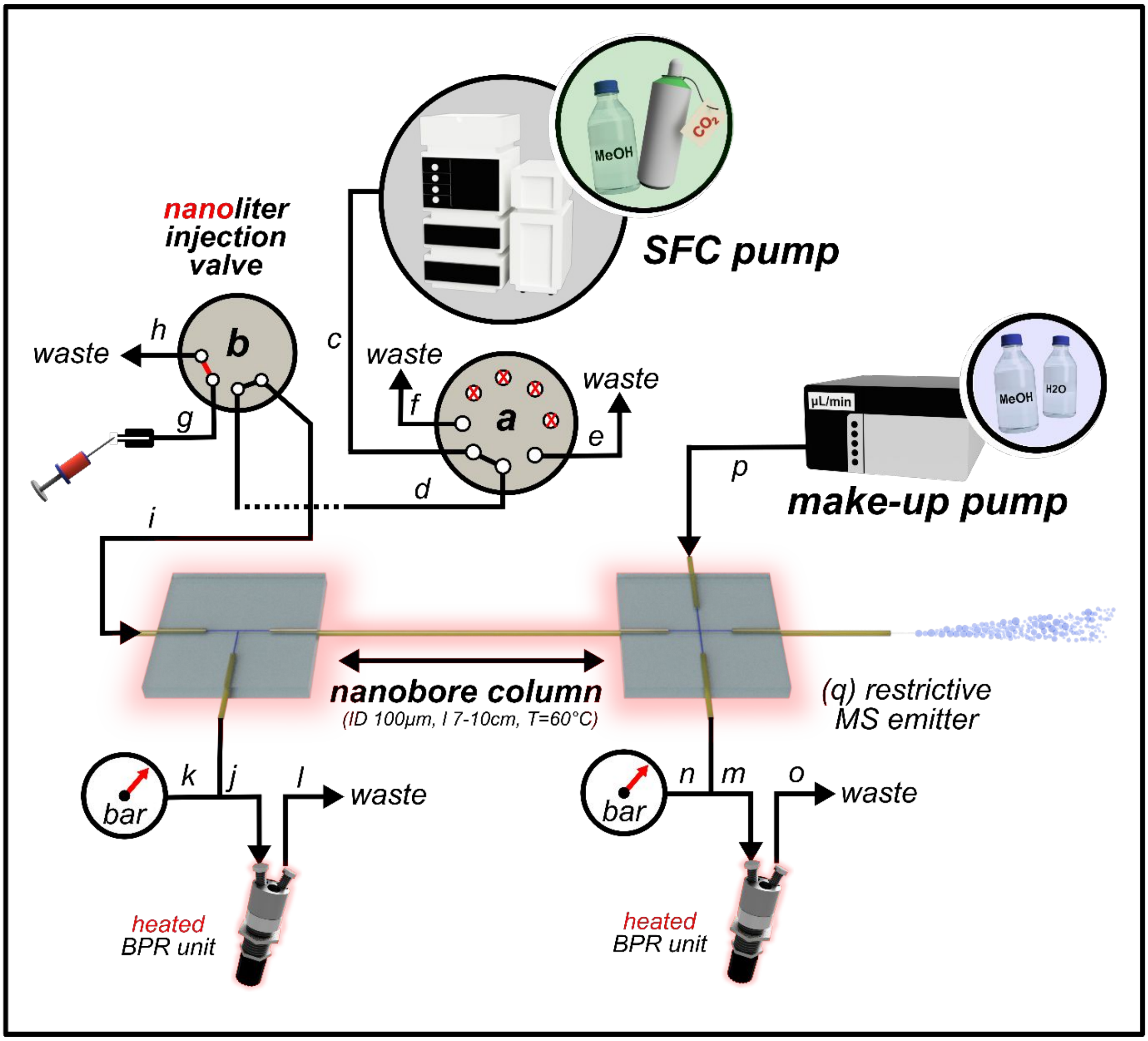

Figure S3 – Detailed schematic overview of the developed modular nanoSFC MS. Parts of the fluidic circuitry are listed in the following. (a) 8-port valve (Cheminert, VICI, Switzerland), (b) nano volume injection valve (5nL injection volume, C74MPKH-4674, VICI, Switzerland), (c) PEEK, OD 1/16, ID 180µm, 50cm, (d) PEEK, OD 1/16, ID 100µm, 30cm and OD 360µm, ID 100µm, 20cm, (e and f) restriction capillary (Agilent G1312-67500), (g) PEEK, OD 360µm, ID 100µm, 20cm, (h) PEEK, OD 360µm, ID 125µm, 15cm, (i) PEEK, OD 360µm, ID 50µm, 15cm, (j) 2x PEEK, OD 360µm, ID 100µm, 20cm + SST OD 1/16, ID 120µm, 12.5cm, (k) PEEK, OD 360µm, ID 100µm, 15cm, (l) SST, OD 1/16, ID 200µm, 12.5cm, (m) 2x PEEK, OD 360µm, ID 100µm, 30cm and PEEK OD 1/16, ID 120µm, 10cm, (n) PEEK, OD 360µm, ID 100µm, 15cm, (o) PEEK, OD 1/16, ID 130µm, 20cm, (p) PEEK, OD 1/16, ID 130µm, 20cm + PEEK, OD 360µm, ID 100µm, 20cm, (q) various types of restrictive MS emitters (made of fused silica, tapered tip, #1 ID 20µm or #2 ID 10µm, both with various lengths). Additionally, two pressure sensors (Duratec, Germany) ensure pressure metering. Two heated BPR units are used to adjust pre- and post-column pressures (static BPR, 1000psi and dynamic SFC BPR; 1300-4200psi, connected via PEEK, OD 1/16, ID 130µm, 20cm). The BPR unit located in the pre-column region used a heater cartridge (Caloratherm, Selerity Technologies, USA) as an unlimited heat source. The pressure BPR unit located in the post-column region was placed into a beaker filled with IPA onto a hot plate (VMR, Germany).

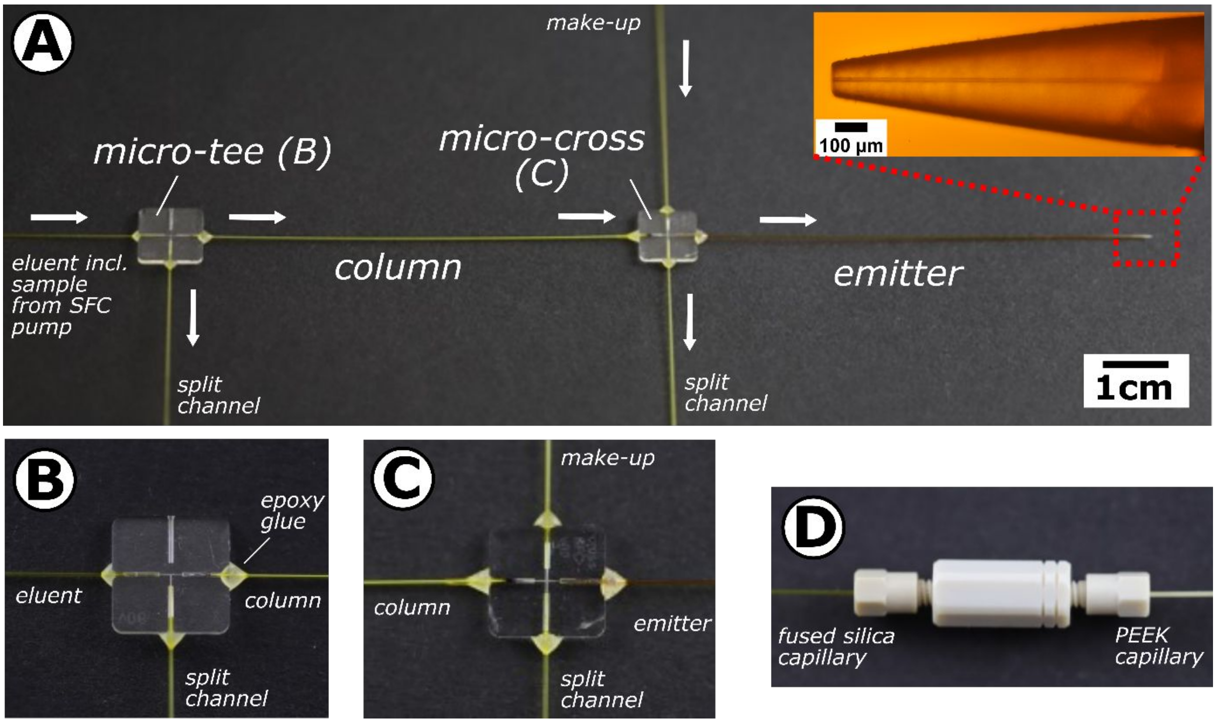

Figure S4 – Photographic representations of the developed modular nanoSFC. The modular nanoSFC can be seen in (**A**). The arrows indicate the direction of the flow. The system consists of two chip-based modules. (**B**) A micro-tee (length 10 x width 10 x depth 1mm) for injection and (**C**) a micro-cross (length 9 x width 9 x depth 1mm) for post-column splitting. The restrictive MS emitter (ID 10µm) with a tapered tip (see insight of A) is connected to the tapered emitter channel of the micro-cross module. The packed nano-bore column is located between both modules. The column and all other capillaries are glued into the chip modules. (**D**) The fluidic connection to the periphery is realized by connecting the fused silica capillaries of the nanoSFC with the PEEK capillaries from the fluidic periphery via a PEEK union (VICI, OD 360µm, bore size 100µm) and two fittings (VICI, OD 360µm).

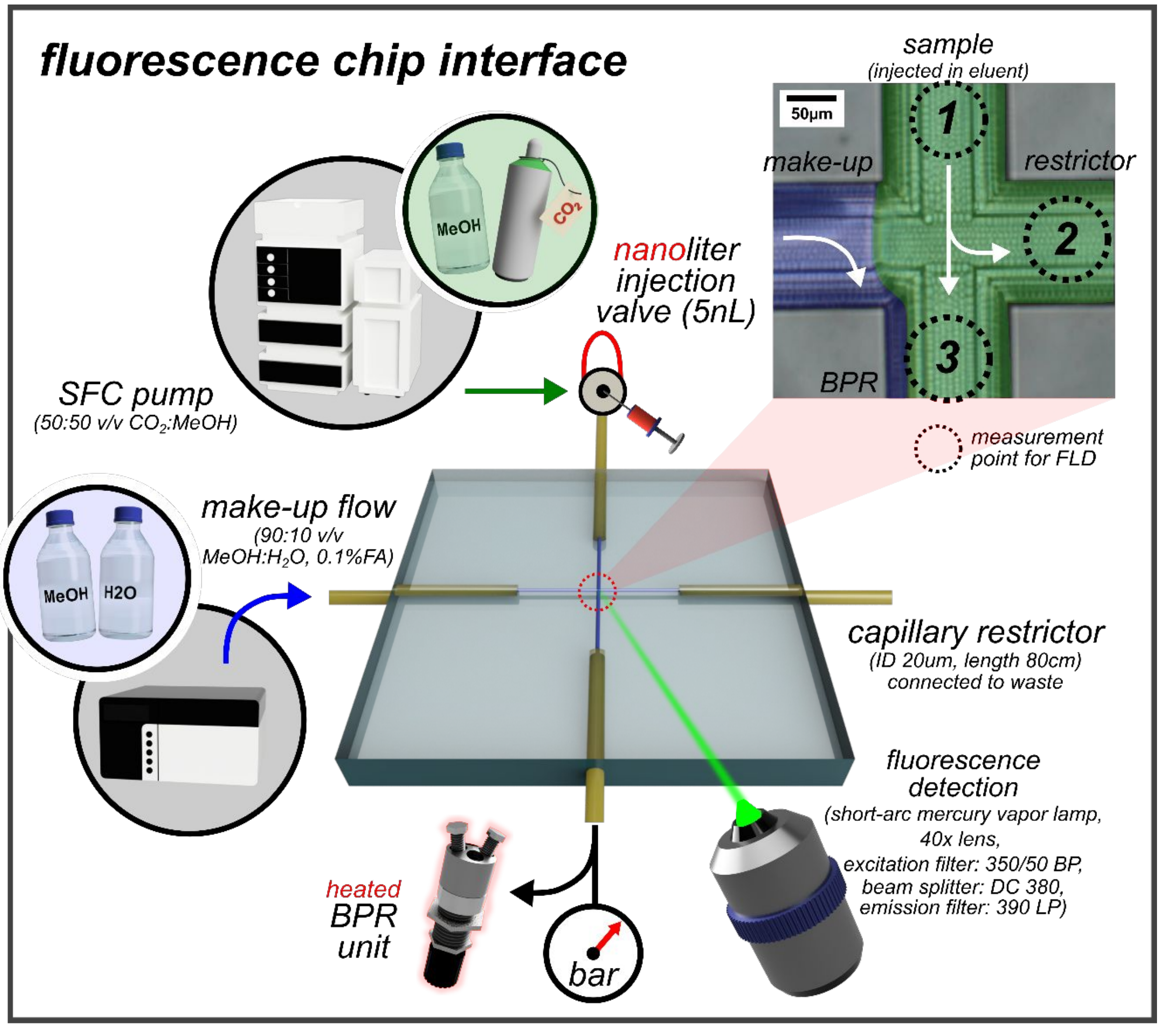

Figure S5 – Instrumental setup used for microscopic and fluorescence inspection of the micro-cross chip module. The insight view displays the detection spots for the fluorescence measurements for the investigation of the peak integrity. Fluidic elements and sensors are listed in the following. (a) nano volume injection valve (5nL injection volume, VICI, Switzerland), (b) PEEK, OD 360µm, ID 50µm, 15cm, (c) PEEK, OD 1/16, ID 130µm, 20cm + PEEK, OD 360µm, ID 100µm, 20cm, (d) (j) 2x PEEK, OD 360µm, ID 100µm, 20cm + SST OD 1/16, ID 120µm, 12.5cm, (e) PEEK, OD 360µm, ID 100µm, 15cm. The BPR unit (static BPR, 1000psi and dynamic SFC BPR; 1300-4200psi, both connected via PEEK, OD 1/16, ID 130µm, 20cm)) used a heater cartridge (Caloratherm, Selerity Technologies, USA) as an unlimited heat source. The pressure sensor was similar to the one used in the setup Figure S2. The fluorescence setup was based on an inverted epifluorescence microscope (IX-71, Olympus, Japan) equipped with a short-arc mercury vapor lamp (Osram HBO 101 W), a 40x lens (LUCPlanFLN, Olympus, Japan), an excitation filter (bandpass 350/50 nm), a dichroic mirror (at 380nm) and an emission filter (long pass 390nm). Detection occurred by a side-on photomultiplier tube (H9305-03, Hamamatsu, Japan) connected to an amplifier.

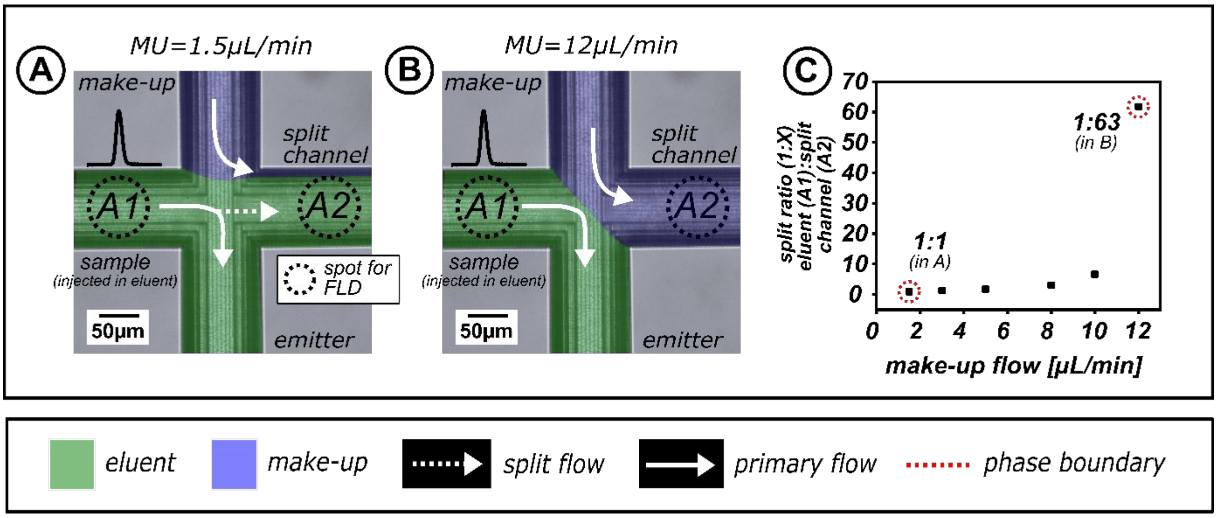

Figure S6 – Split behavior of micro-cross chip module. Microscopic view of the micro-cross under different make-up flow conditions: (**A**) 1.5µL/min, split channel open and (**B**) 12µL/min, split channel closed. (**C**) Relationship between the applied make-up flow and split ratio. The split ratios were determined by measuring the peak area of a fluorescent sample plug under varying make-up flow conditions at the entrance to the micro-cross (A1) and at the split channel (A2). The decreasing split ratio is caused by the make-up flow constricting the split channel. The closure of the split channel prevents access to the back-pressure regulator, eliminating pressure damping and causing segmentation of signal peaks. The segmentation of the signal peak can be remedied by introducing a column in front of the micro-cross structure.

The microscopic images show the dynamic nature of the fluidic steady state between the eluent (green, 50:50 v/v CO_2:MeOH) and the make-up flow (blue, 90:10 v/v MeOH:H_2O, 0.1%FA). Increasing make-up flow or lowering inlet pressure changed the position of the phase boundary (red dotted line) and reduced eluent proportion (illustrated by the dotted arrow) within the split channel. If a splitless interface is created where the eluent cannot access the split channel, sample segmentation was observed in the instrumental setup (Figure S4).

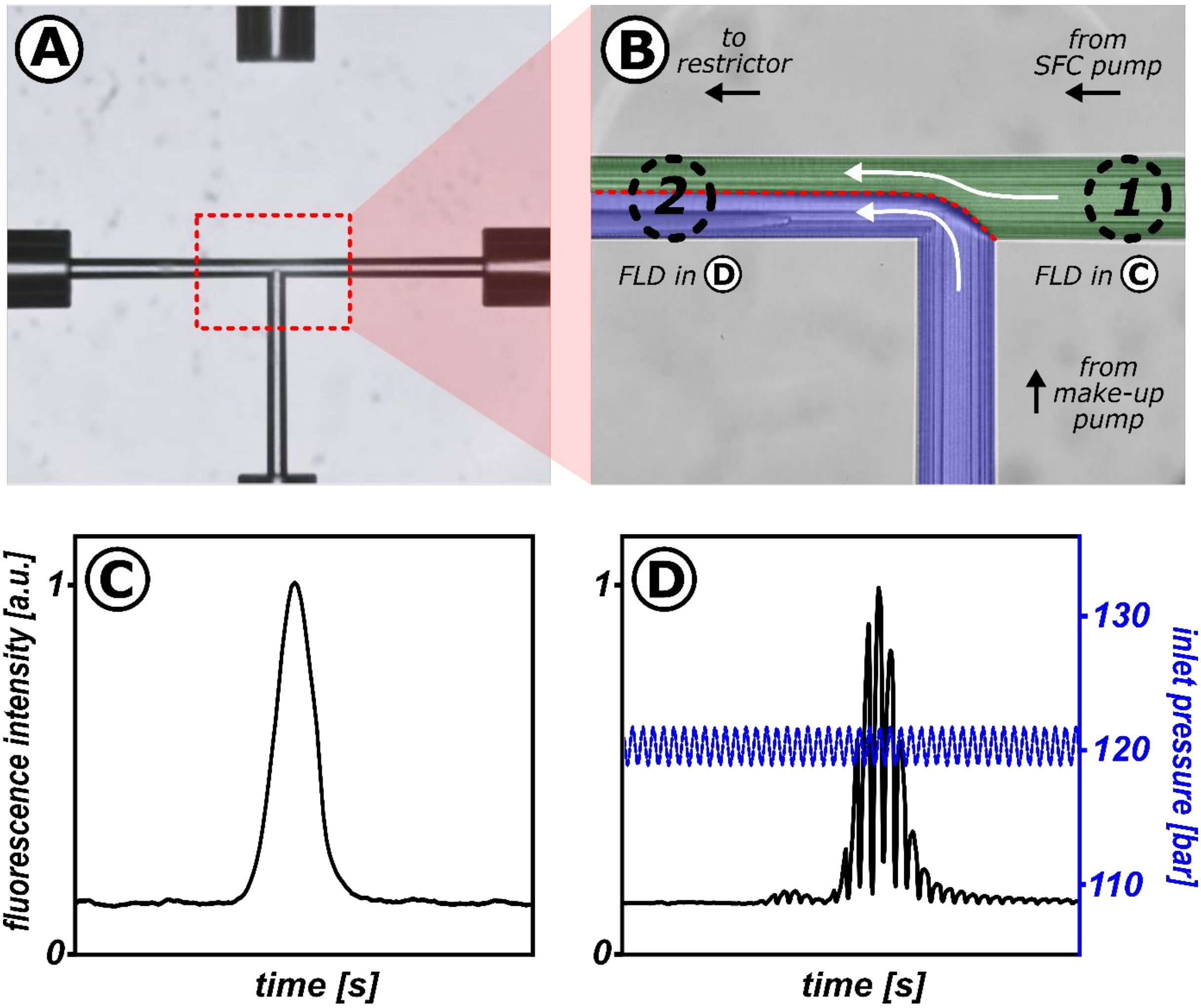

Figure S7 – Sample segmentation under splitless conditions. Therefore, a micro-tee chip module (**A**) using fluorescence detection was evaluated. Its fluidic insight is illustrated in (**B**). Herein, CO_2-containing eluent (green, 50:50 v/v CO_2:MeOH, p=120bar) is joined by a viscous make-up stream (blue, 90:10 v/v MeOH:H_2O; 0.1%FA, F=5µL/min) at the micro-tee-junction (ID 100µm) to confluence into the restrictor (ID20µm, l=80cm). This is indicated by the clear presence of the phase boundary (red dotted line). Fluorescence detection of a sample plug (c=100µM c120 dissolved in MeOH) was conducted before (1) and after the micro-tee junction (2) using the instrumental setup described in Figure S4, with the exception of a backpressure-stabilized split channel. The detected fluorescent sample plug remained intact and unsegmented before the micro-tee junction (**C**). After the micro-tee junction, the integrity of the sample plug was not given, as illustrated by the segmentation seen in (**D**). This sample segmentation is a result of the missing pressure-damping effect within the micro-tee structure. The observations made confirmed those made in figure 4B in the main manuscript, in which the split channel of the micro-cross is not accessible by the eluent stream.

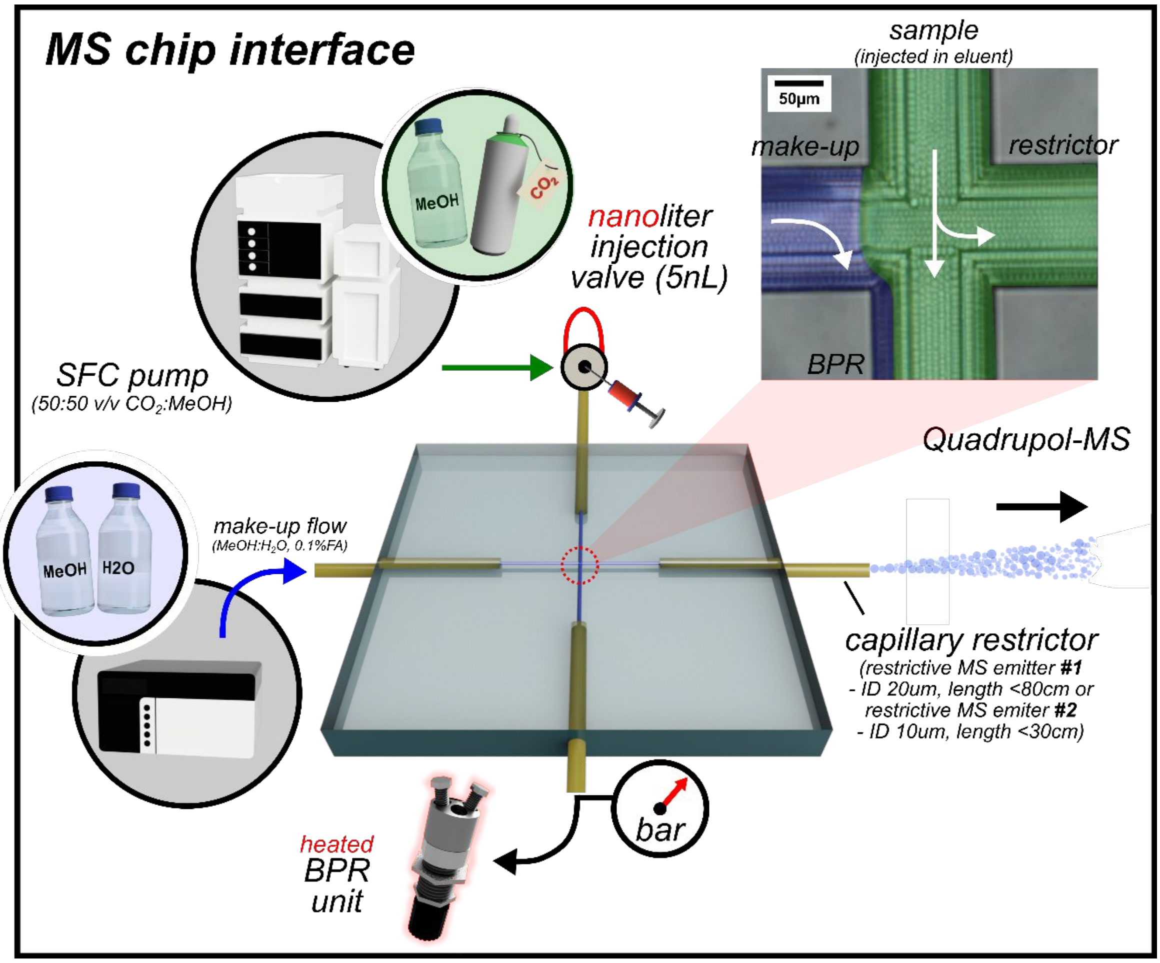

Figure S8 - Instrumental setup used for mass spectrometric evaluation of the micro-cross chip module. Fluidic elements and sensors are similar to the instrumental setup described in Figure S3, with the exception that the installed capillary restrictor was used as an MS emitter instead. MS emitters with IDs of 10 and 20µm (in various lengths) were tested to simulate different mass flow conditions. The different micro-cross configurations (exemplarily the eluent 90° to emitter configuration shown in the insight view) allow investigating the make-up flow's impact on MS sensitivity.

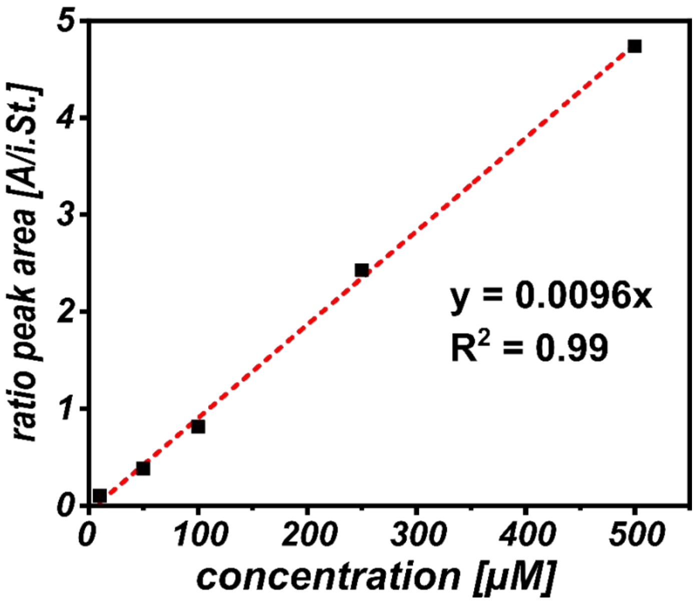

Figure S9 – Suitability of an internal standard to determine the MS sensitivity for the micro-cross chip module. Samples of different DL-α-tocopherol concentrations (labeled A) were doped with ergocalciferol (c=1mM, labeled i.St.). The subsequent mixture was injected into the micro-cross chip module and detected using MS. Calculating the quotient (A/i.St.) from the peak areas of both MS signals, a linear relationship could be determined over the analyte concentration range investigated. This indicates that both compounds behave similarly and allow an unbiased determination of the MS sensitivity. Instrumental conditions: mobile phase: 90:10 v/v CO_2:MeOH, inlet pressure 120bar, no column was used, make-up flow: 90:10 v/v MeOH:H_2O; 0.1%FA, 1.5µL/min, restrictor: ID20µm, 80cm, blunt end. MS parameter: capillary voltage: -4kV, positive ion mode, SIM 473*m/z* and 397.5*m/z*, fragmentor 300V and 275V, 4Hz

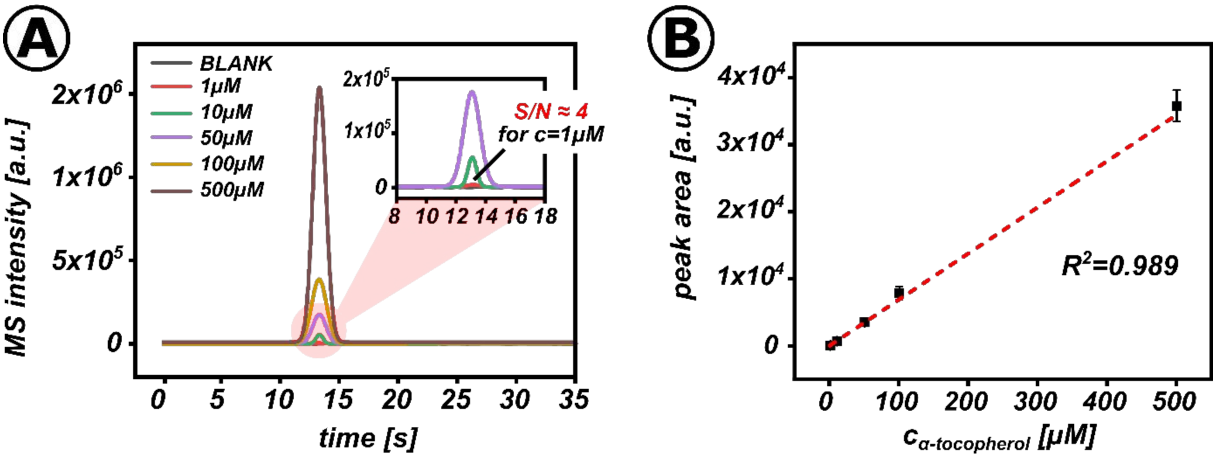

Figure S10 – Low micromolar sensitivity of modular, chip-based nanoSFC-MS illustrated by (**A**) SIM-chromatograms of on-column injection of DL-α-tocopherol with increasing concentrations (1-500µM) and (**B**) the resulting calibration curve, instrumental parameters: eluent: 90:10 v/v CO_2:MeOH, column: ID100µm, *l* 7cm, 2-EP, dp=5µm, make-up: 1.5µL/min MeOH:H_2O; 0.1% FA, cross configuration: eluent 180° to emitter, emitter ID 10µm, length 7cm, t=60°C, inlet p=140bar, outlet p=98bar, acquisition rate: 8Hz, DL-α-tocopherol samples are dissolved in MeOH. MeOH served as a blank, peak smoothing was applied. Three injections were carried out for each concentration. Six injections for the blank value.

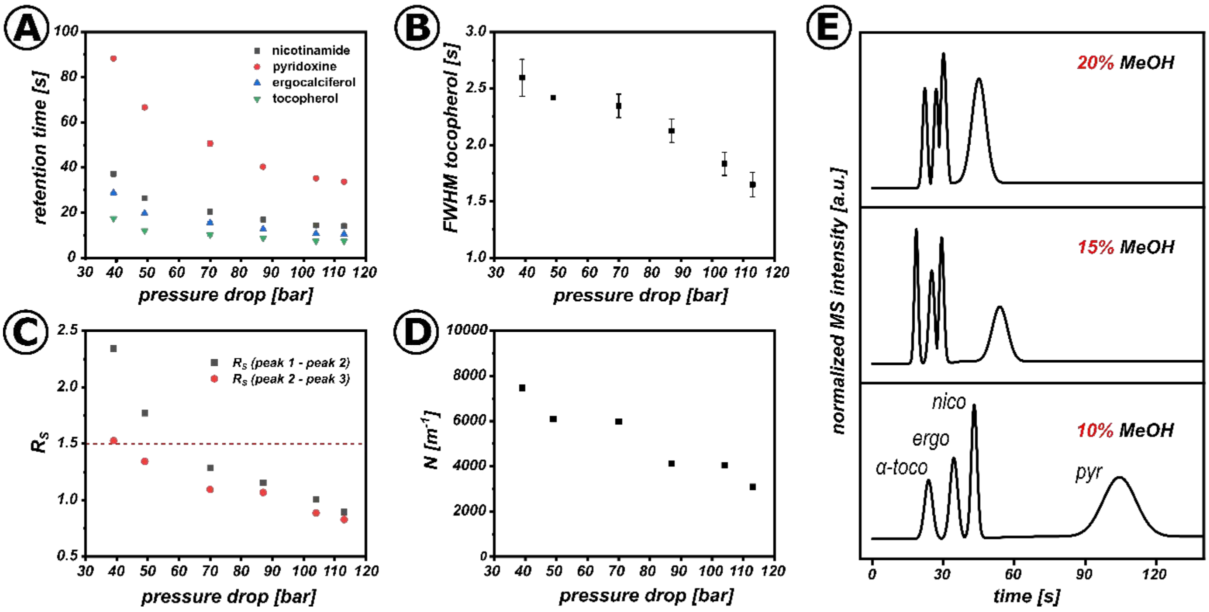

Figure S11 – Influence of pressure drop and modifier content on different chromatographic parameters of the achiral separation. The impact of the pressure drop on (**A**) retention time, (**B**) peak width as full width at half maximum (FWHM), (**C**) peak resolution (R_S) and (**D**) separation efficiency. (**E**) Influence of different modifier proportion (10, 15, 20% v/v MeOH) on the achiral separation

Instrumental parameter used for the pressure drop experiments (**A-D**): eluent: 90:10 v/v CO_2:MeOH with varying modifier proportion, maximal inlet pressure: 220bar column: 2-EP, dp=5µm, l=7cm, make-up: 90:10 v/v MeOH:H_2O, 0.1%FA, emitter: ID 10µm, l=7cm, post-column configuration: eluent 180° to emitter, The pressure drop was calculated from the difference between the pre and post-column pressures. Instrumental parameter used for the modifier experiments (**E**): inlet p=120bar, column: 2-EP, dp=5µm, l=8cm, make-up: 90:10 v/v MeOH:H_2O, 0.1%FA, F=1.5µL/min, emitter: ID 10µm, l=7cm, post-column configuration: eluent 180° to emitter, MS parameter: position ion mode, capillary voltage: -4kV, dry gas: 1 l/min N_2 at 200°C, acquisition rate: 4Hz, sample: 1mM of DL-α-tocopherol (α-toco), ergocalciferol (ergo), nicotinamide (nico) and pyridoxine (pyr) dissolved in MeOH.
